# Transcriptomic profiles reveal differences between the right and left ventricle in normoxia and hypoxia

**DOI:** 10.14814/phy2.14344

**Published:** 2020-01-20

**Authors:** Matthew W. Gorr, Krishna Sriram, Amy M. Chinn, Abinaya Muthusamy, Paul A. Insel

**Affiliations:** ^1^ Dorothy M. Davis Heart and Lung Research Institute College of Medicine The Ohio State University Columbus OH USA; ^2^ College of Nursing The Ohio State University Columbus OH USA; ^3^ Department of Pharmacology University of California San Diego La Jolla CA USA; ^4^ Department of Medicine University of California San Diego La Jolla CA USA

**Keywords:** hypoxia, immune cells, pulmonary hypertension, remodeling, right ventricle, transcriptomics

## Abstract

Chronic hypoxia from diseases in the lung, such as pulmonary hypertension, pulmonary fibrosis, and chronic obstructive pulmonary disease, can increase pulmonary vascular resistance, resulting in hypertrophy and dysfunction of the right ventricle (RV). In order to obtain insight into RV biology and perhaps uncover potentially novel therapeutic approaches for RV dysfunction, we performed RNA‐sequencing (RNA‐seq) of RV and LV tissue from rats in normal ambient conditions or subjected to hypoxia (10% O_2_) for 2 weeks. Gene ontology and pathway analysis of the RV and LV revealed multiple transcriptomic differences, in particular increased expression in the RV of genes related to immune function in both normoxia and hypoxia. Immune cell profiling by flow cytometry of cardiac digests revealed that in both conditions, the RV had a larger percentage than the LV of double‐positive CD45^+^/CD11b/c^+^ cells (which are predominantly macrophages and dendritic cells). Analysis of gene expression changes under hypoxic conditions identified multiple pathways that may contribute to hypoxia‐induced changes in the RV, including increased expression of genes related to cell mitosis/proliferation and decreased expression of genes related to metabolic processes. Together, the findings indicate that the RV differs from the LV with respect to content of immune cells and expression of certain genes, thus suggesting the two ventricles differ in aspects of pathophysiology and in potential therapeutic targets for RV dysfunction.

## Graphical abstract

The right ventricle (RV) of the heart is affected with many pulmonary disorders including pulmonary hypertension. Here, we use RNA‐seq. to characterize the altered genes in the RV and the left ventricle (LV) during hypoxia in a rat model. We compare changes in gene expression, but most importantly find a difference in immune cell‐related genes in the RV compared to the LV, and confirm this using flow cytometry and immunoblotting.

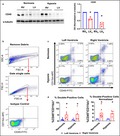

## 1 INTRODUCTION

Chronic hypoxia in diseases such as pulmonary hypertension, chronic obstructive pulmonary disease, and pulmonary fibrosis can lead to increased pulmonary vascular resistance and result in increased afterload to the right ventricle (RV) (Lettieri, Nathan, Barnett, Ahmad, & Shorr, 2006; Ryan & Archer, 2014; Terzano et al., 2010). Similar to the pathobiology of the left ventricle (LV), this compensatory action by the RV leads to hypertrophy, which may then lead to RV failure (Friedberg & Redington, 2014). Although the biology of the RV is generally considered to be identical to that of the LV, RV failure has a poorer prognosis and response to therapy compared to LV disease, due in part to factors independent of the increase in pulmonary arterial (PA) pressure (Ghio et al., 2001). These unexplained factors are manifested as disproportional levels of fibrosis, alterations in metabolism, and RV ischemia (Ryan & Archer, 2014). RV failure is also a strong indicator of poor prognosis is patients with LV failure (Ghio et al., 2001; Meyer et al., 2010). No specific therapies target the RV and current therapeutic strategies for diseases causing RV failure lack efficacy.

Transcriptomic analysis using RNA sequencing (RNA‐seq) is an unbiased approach to define gene expression and may offer a means to study the pathobiology of RV disease and uncover new mechanisms that might help identify novel RV‐specific therapies. Animal models, such as chronic hypoxia, can mimic the RV remodeling in humans in response to increased PA pressure. Here, we utilized RNA‐seq to compare the RV and the LV of Sprague Dawley rats in normoxia and hypoxia. Analyses of the RNA‐seq data revealed differentially expressed genes in pathways not previously invoked in RV biology or dysfunction. Notably, the RV selectively expressed genes related to immune function. Immunoblotting, and flow cytometry revealed that the RV contains an increased number of immune cells. Thus, the RV and LV differ in their gene expression profiles and unexpectedly, in their expression of certain cell types, in particular cells involved in immune function.

## 2 METHODS

### 2.1 Development of RV hypertrophy in rats

All animal procedures were approved by the University of California San Diego (UCSD) Institutional Animal Care and Use Committee. Adult male rats (3–4 mo. old) were housed in a temperature‐controlled environment with a 12 hr light/dark cycle. Rats were subjected to hypoxia (10% O_2_) in a hypobaric chamber or were housed in room air as a normoxic control. After 2 weeks, rats were anesthetized with ketamine and xylazine (100 and 10 mg/kg, respectively) and sacrificed by removing the heart.

### 2.2 RNA isolation from cardiac tissue and RNA‐seq

Hearts were washed with phosphate‐buffered saline (PBS), the atria were removed, and the RV was separated from the LV and weighed. The LV and RV were snap‐frozen in liquid nitrogen and later digested in lysis buffer (Buffer RLT, QIAGEN). RNA was isolated with an RNeasy Mini Kit (QIAGEN) from the LV and RV free walls (*n* = 6 rats for a total of 12 samples). RNA‐seq was conducted at the UCSD Institute for Genomics Center. Libraries were prepared for sequencing of polyadenylated RNA via the TruSeq stranded mRNA protocol (Illumina) and sequenced on an Illumina HiSeq 4000, with samples sequenced at ~30 million 50 bp single reads, on average.

### 2.3 RNA‐seq analyses

Following standard quality control steps on Galaxy (Afgan et al., 2016), FASTQ files were analyzed by Kallisto Bray, Pimentel, Melsted, and Pachter (2016) using Ensembl reference transcriptome for alignment. Transcript expression from Kallisto was converted to gene‐level expression (in transcripts per million (TPM) and estimated counts) via the Tximport package (Soneson, Love, & Robinson, 2015). Estimated counts were used as input to the edgeR package (Robinson, McCarthy, & Smyth, 2010); genes having differential expression (DE) with a false discovery rate (FDR) <0.05 were considered statistically significant. Analysis for gene ontology and other associations with annotated gene sets was conducted with Enrichr (Kuleshov et al., 2016), as described below. Sets of significantly up‐regulated or down‐regulated (FDR < 0.05) genes were separately queried to test the likelihood if certain annotated sets of genes were statistically overrepresented among differentially expressed genes. We evaluated results from Enrichr for Gene Ontology (GO) annotated sets of genes based on a) the biological processes in which they participate, b) the molecular function of their gene products in a cell and c) the cellular compartment with which they are associated with (Mi, Muruganujan, Ebert, Huang, & Thomas, 2019). We also used Enrichr to evaluate associations with certain cell types, based on annotations from the BioGPS database (Wu et al., 2009), which allows one to test whether differentially expressed genes are strongly associated with a particular cell type. Enrichr was then used to test for enrichment of DE genes for specific signaling pathways, based on annotations from the Reactome database (Fabregat et al., 2018). In addition, we tested (via Enrichr) associations of DE genes with specific cellular compartments based on their annotations via the Jensen database (Binder et al., 2014), yielding complementary results for those from the GO cellular compartment analysis. For all analyses via Enrichr, associations were considered statistically significant if they had *q*‐values (i.e., *p*‐values adjusted for multiple testing) <.05.

We used STRING (https://string-db.org/) (Szklarczyk et al., 2015) to visualize networks of DE genes. Default settings (except for hiding unconnected nodes and disabling structure previews) and genes corresponding to highlighted pathways or processes were used as to provide a visual representation of closely associated, simultaneously up‐ or down‐regulated sets of genes.

We also performed gene set enrichment analysis (GSEA) (Subramanian et al., 2005), in which we ranked genes with DE and FDR < 0.05 by fold‐change; negative values were used for reduced expression. GSEA analysis was conducted by using pre‐ranked analysis, with the combined GO gene sets (collection C5 in the MSigDB database curated by the Broad institute, (http://software.broadinstitute.org/gsea/msigdb/index.jsp), which yields a combined enrichment analysis by using the GO annotations for biological processes, molecular function and cellular compartment. One obtains information regarding which annotated gene sets are enriched. The GSEA analysis was thus complementary to and helped confirm findings from analysis via Enrichr, as shown in Results. GSEA‐weighted analysis of genes that are up‐ and down‐regulated confirmed that enrichment for gene sets was specific to those genes that were either up‐ or downregulated, but not both, since such a result would yield net zero enrichment. Gene sets were considered significantly enriched if they had an FDR < 0.05.

Following the GSEA preranked analysis, we performed (via the GSEA software) leading edge analysis on significantly enriched gene sets to identify genes that are members of multiple enriched gene sets. Such genes likely play a role in multiple pathways/processes that are up‐regulated and potentially may be nodal elements in the network of genes dysregulated in hypoxia in this study.

### 2.4 Access to RNA‐seq data

Data have been deposited at NCBI’s Gene Expression Omnibus (GEO) with accession number http://www.ncbi.nlm.nih.gov/geo/query/acc.cgi?acc=GSE133402.

### 2.5 Immunoblotting

Pieces of RV and LV tissue were lysed in RIPA buffer and then flash‐frozen. Lysates were electrophoresed on SDS‐PAGE gels, transferred to a nitrocellulose membrane, and blocked with 5% milk in TBS with 0.05% TWEEN®‐20 (TBST). Blots were incubated overnight with anti‐CD45 antibody (Abcam, 10558) or anti‐α‐tubulin (Abcam, MS‐581‐P1ABX), then washed with TBST and incubated with anti‐rabbit HRP (GE Healthcare, NA9340V, for CD45) or anti‐mouse HRP (GE Healthcare, NXA931V, for α‐tubulin) for 1 hr at room temperature. Blots were washed again and developed with ECL substrate (Thermo Fisher Scientific), and imaged on a digital imager.

### 2.6 Immunohistochemistry

Intact heart slices were frozen in optimal cutting temperature (OCT) compound (Tissue‐Tek) and sectioned on a cryostat. Sections were fixed in cold methanol for 20 min, washed with PBS, and stained with hematoxylin and eosin (H&E, Merck KGaA and Fisher Scientific, respectively) or Masson's Trichrome (Abcam).

### 2.7 Flow cytometry

We digested cardiac tissue from a subset of rats in the hypoxic and normoxic groups to obtain a single‐cell suspension for flow cytometry. Rats were anesthetized as indicated above, the heart was flushed with PBS, and the RV and LV free walls were separated. Tissue was cut into ~1 mm^3^ pieces and placed in PBS with collagenase II (150 U/ml, Worthington Biochemical Co., CLS‐2) and incubated for 1 hr at 37°C. Tissue was then triturated and filtered through a 40 μm filter and centrifuged at 250*g* for 8 min. The resulting pellet was resuspended in FACS buffer (PBS with 0.5% BSA and 1 mM EDTA) and stained with antibodies directed against rat CD45‐FITC (Biolegend 202205), CD11b/c‐APC (Biolegend 201809) (leukocyte surface markers) or the respective isotype controls (Biolegend 400107 and 400219) for 20 min, followed by washing with FACS buffer. Single‐stain groups were used for compensation, a method that corrects for the spectral overlap between the different emission spectra of fluorochromes. We conducted flow cytometry in each sample on at least 50,000 events using the BD FACSCanto II (BD Biosciences). Data were analyzed using FlowLogic (Miltenyi Biotec).

## 3 RESULTS

### 3.1 Induction of RV hypertrophy by hypoxia

**Figure 1:**
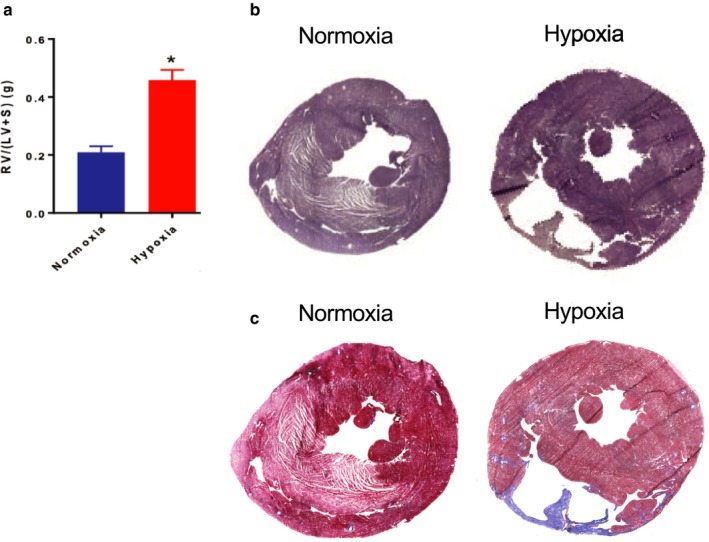
Hypoxia induces right ventricle (RV) remodeling. (a) Fulton index [RV weight/ (LV + septum weight)], *n* = 3, *p<.05 via *t*‐test. Representative staining from hypoxia and normoxia‐treated rats (*n* = 3) shown for (b) H&E and (c) Masson's Trichrome

We isolated cardiac tissue from adult rats subjected to hypoxia or normoxia for 2 weeks. The RV weight/ (LV + septum) weight (Fulton index) was significantly increased in rats exposed to hypoxia (Figure 1a, *n* = 3, Student's *t*‐test *p* < .001). H&E staining of tissue sections revealed an increase in RV chamber thickness and size, and in some portions, thinning of the RV free wall (Figure 1b). Trichrome (blue) staining demonstrated fibrosis in the RV during hypoxia (Figure 1c). Thus, 2 weeks of hypoxia induced significant RV hypertrophy and fibrosis.

### 3.2 The RV expresses a distinct set of genes compared to that of the LV in rats exposed to normoxia or hypoxia

**Figure 2:**
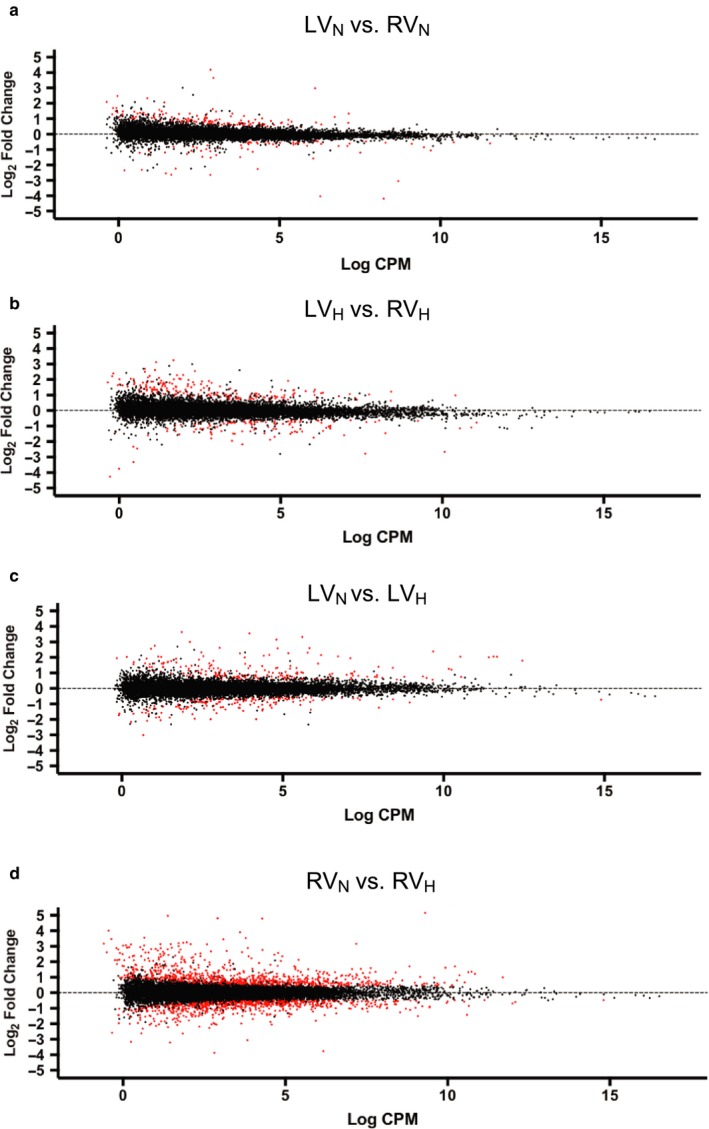
RNA‐seq analysis of rat cardiac tissue. Smear plots show log_2_ fold change versus log_2_ counts per million (CPM) for each differentially expressed gene. Red dots indicate genes with FDR < 0.05. Analyses are shown for: (a) LV in normoxia (LV_N_) versus RV in normoxia (RV_N_), (b) LV in hypoxia (LV_H_) versus RV in hypoxia (RV_H_), (c) LV_N_ versus LV_H_, and (d) RV_N_ versus RV_H_

**Figure 3:**
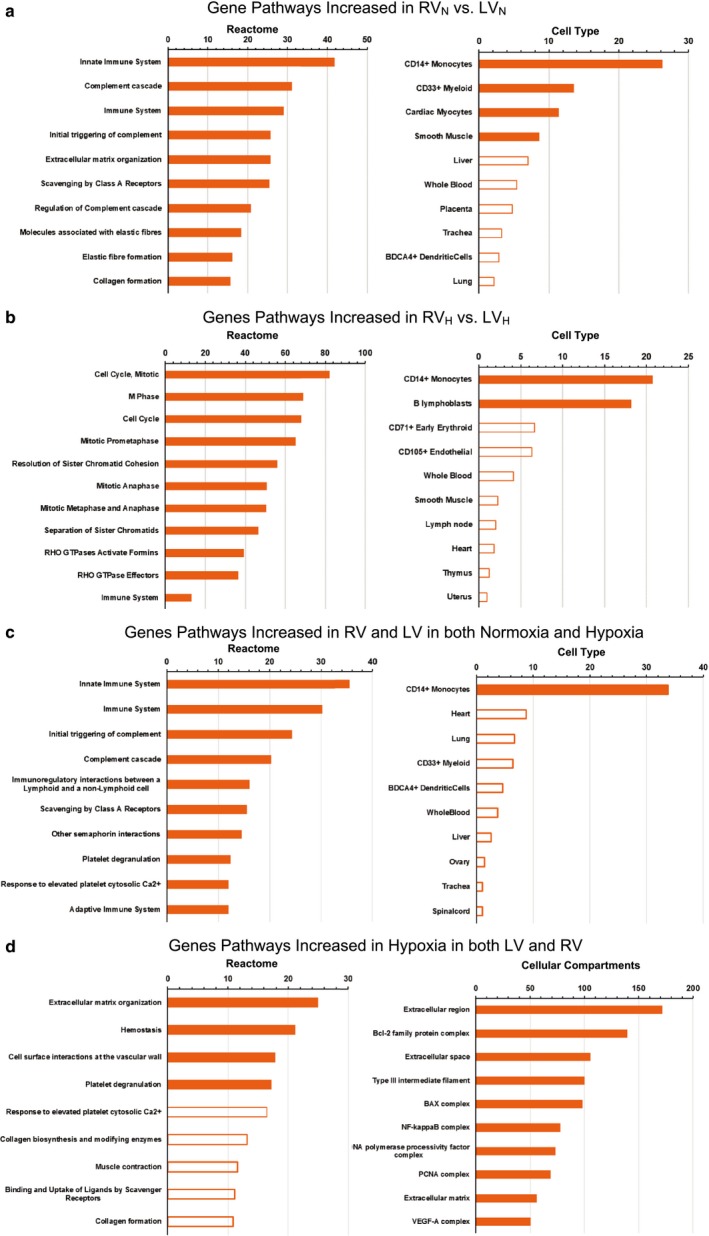
Curated Reactome pathways, cell types and cell compartments. BioGPS was used to analyze up‐regulated genes (with an FDR < 0.05) in comparisons of: (a) RV in normoxia (RV_N_) versus LV in normoxia (LV_N_), (b) RV in hypoxia (RV_H_) versus LV in hypoxia (LV_H_), (c) pathways increased in RV and LV in both Normoxia and Hypoxia, and (d) pathways increased in hypoxia in both LV and RV

We used RNA‐seq to assess differences in gene expression between the RV and LV in normoxia (RV_N_ and LV_N_) and hypoxia (RV_H_ and LV_H_). The ventricles expressed a similar number of transcripts in normoxia (RV_N_: 16,495 ± 301; LV_N_: 16,104 ± 277) and hypoxia (RV_H_: 16,634 ± 282; LV_H_: 16,320 ± 148) (mean ± *SD*, *n* = 3; ANOVA, *p* > .10). DE analysis (using edgeR) revealed 138 genes whose expression was increased and 70 genes with decreased expression (with a false discovery rate [FDR] <0.05) in the RV_N_ group compared to the LV_N_ group (Figure 2a, Table S1) With hypoxia, 203 genes had increased expression and 115 genes had decreased expression in the RV_H_ group compared to the LV_H_ group (Figure 2b, Table S2). Enriched pathways (*P* adjusted < 0.05) among the DE genes were identified utilizing Enrichr. Figure 3 shows the curated Reactome pathways, cell types and cell compartments based on analysis by BioGPS. Under normoxic conditions, the RV had increased expression of genes associated with the immune system; of note, CD14^+^ (monocyte marker) and CD33^+^ (Siglec‐3 [sialic acid binding Ig‐like lectin 3], myeloid cell lineage marker) cells were the predominantly enriched cell types in the RV (Figure 3a). Similar results were found using STRING analysis, in which the RV expresses distinct clusters of genes for antigen presentation, innate immunity and ECM organization (Figure S1). Comparison of the RV to the LV in hypoxia revealed that pathways related to cell growth and mitosis were highly enriched, but several immune cell types, including CD14^+^ cells, were also enriched in the RV (Figure 3b, Figure S2). Analysis of RV‐specific pathways (i.e., genes increased in the RV in both normoxia and hypoxia) identified RV‐specific pathways relating to immune cell function. CD14^+^ monocytes were the only cell type significantly enriched in the RV (Figure 3c).

### 3.3 Hypoxia alters the expression of many genes in the RV and LV

Comparison of the RV_H_ group to the RV_N_ group identified 1,012 genes whose expression was increased and 845 genes whose expression was decreased (FDR < 0.05, Figure 2c, Table S3). By contrast, 222 genes had increased expression and 160 genes had decreased expression in the LV_H_ compared to the LV_N_ group (FDR < 0.05, Figure 2d, Table S4). Certain genes had altered expression with hypoxia in both the RV and the LV: expression of 61 genes was increased and of 49 genes was decreased in both the LV_H_ and RV_H_ groups compared to their normoxic controls. These genes included the gene for natriuretic peptide A (NPPA/ANP), which is up‐regulated in hypoxia by HIF‐1α Chun et al., (2003). Analysis of enriched pathways for genes increased in the LV_H_ and RV_H_ samples indicated an increase in extracellular matrix groups, as observed in both the Reactome and Jensen (Cellular Compartments) databases (Figure 3d).

**Figure 4:**
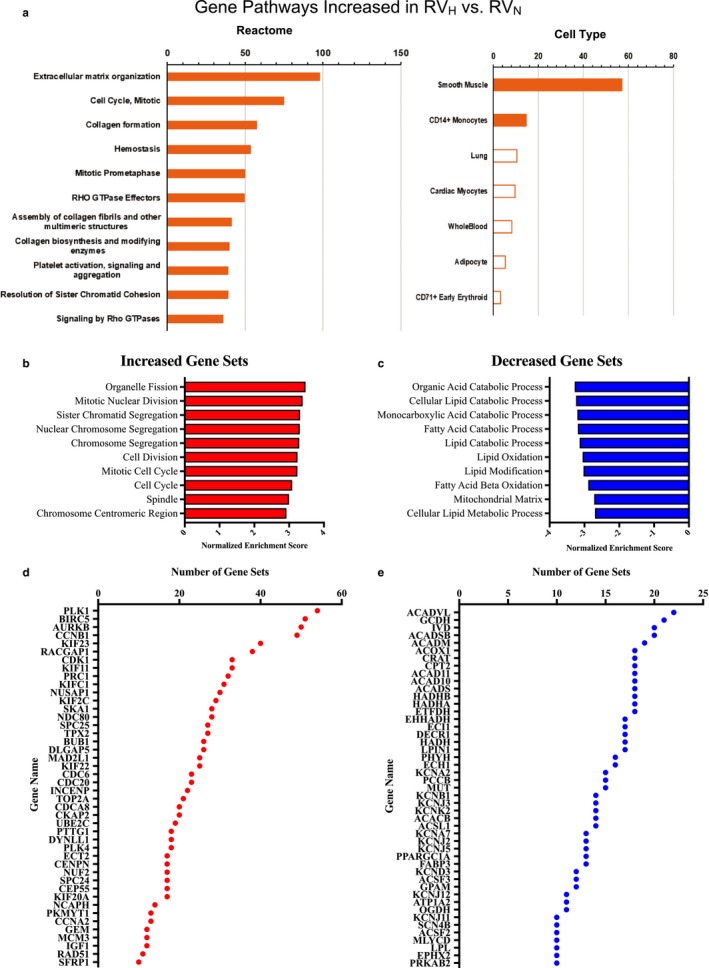
Comparison of gene changes between the RV in hypoxia and the RV in normoxia. (a) Curated Reactome pathways cell type based on analysis by BioGPS and gene set enrichment analysis for genes with DE (and a FDR < 0.05) that are (b) increased (from 69 total genes sets) and (c) decreased (from 56 total gene sets). Leading edge analysis of (d) up‐regulated genes and (e) down‐regulated genes, implicating these genes as potential regulators in parallel of multiple enriched, regulated processes

Gene set enrichment analysis (GSEA) reveals distinct sets of genes involved in multiple pathways in RV hypertrophy. Figure 4a shows the enriched groups (using Enrichr) among DE genes that are increased in the RV with hypoxia. These groups included extracellular matrix organization and pathways relating to cell signaling and mitosis. We also used GSEA to assess DE genes that were increased and decreased in the RV_H_ group compared to the RV_N_ group. Other group comparisons were not analyzed with GSEA due to the lower number of DE genes. Figure 4b and c show respectively the 10 most‐enriched GO gene sets that were increased or decreased. In total, 69 GO gene sets were enriched and up‐regulated, the majority of which involved processes related to the cell cycle and cell division. For genes that were down‐regulated, 56 GO sets showed enrichment, the majority of which involved metabolic processes. The q‐value for all groups shown was ~0, implying that these associations have very high statistical significance. Leading edge analysis of up‐regulated genes revealed that 44 genes are common in ≥10 of the 69 enriched gene sets that are up‐regulated and that 47 genes are common in ≥10 of the 56 enriched down‐regulated gene sets, implicating these genes as potentially parallel regulators of multiple enriched processes (Figure 4d and e).

### 3.4 The RV has more resident immune cells than does the LV

**Figure 5:**
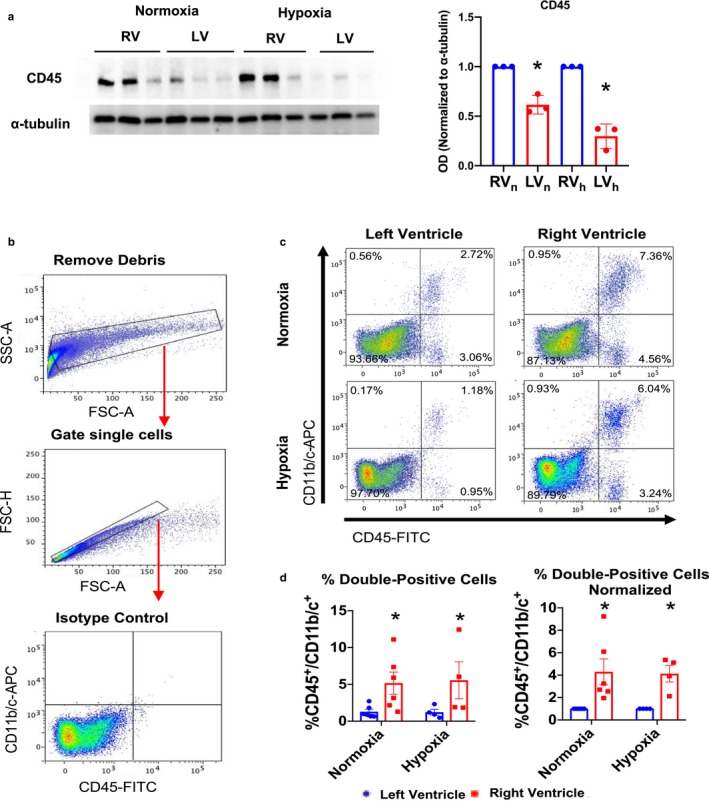
The rat RV contains more immune cells than the LV in normoxia and hypoxia. (a) Representative immunoblot and densitometric calculation for CD‐45 and α‐tubulin (loading control), * indicates *p* < .05 for differences between the LV and RV via ANOVA. (b) Flow cytometry was performed on cardiac digests (*n* = 4–6 per group): Gating strategy and isotype control are shown (c) representative images of CD11b/c‐APC and CD45‐FITC in cardiac samples from flow cytometry; (d) quantitation of the percentage of double‐positive cells in all samples, as assessed by flow cytometry, with data normalized (to results for LV) for each rat. *p < .05 for ventricle effect via two‐way ANOVA

Since we found multiple GO terms that involve immune cell function in the comparison of the RV to the LV, we sought to independently confirm these findings at the protein level by assessing immune cell expression using immunoblotting and immunofluorescence of LV and RV tissue from rats subjected to normoxia or hypoxia. Because RNA‐seq was conducted on whole tissue, the gene expression data could not identify changes in particular immune cell types. We used CD45, a hematopoietic cell marker that detects multiple types of leukocytes (including T cells, B cells, dendritic cells, macrophages/monocytes, and granulocytes), and CD11b/c, which is predominantly a macrophage and dendritic cell marker but can also be present on other leukocytes. Immunoblotting revealed that CD45 expression (a hematopoietic cell marker) was increased in the RV of both normoxia and hypoxia hearts (Figure 5a, *p* < .05 via two‐way ANOVA for ventricle difference, *n* = 3 hearts/group). To obtain more quantitative data, we performed flow cytometry (with gating to remove debris and assess only single cells) to examine the immune cell profile in normoxic and hypoxic LV and RV. We examined expression of the leukocyte surface markers CD11b/c and CD45. We found an increase in the CD45^+^/CD11b/c^+^ population in the RV compared to the LV of rats exposed to normoxia or hypoxia ((*p* < .05 via two‐way ANOVA) but without changes induced by hypoxia. (Figure 5b–d). Analysis of each RV with the LV from the same heart showed that the RV’s had on average, a 4‐fold higher percentage of double‐positive cells (Figure 5d).

## 4 DISCUSSION

These studies revealed that exposure of rats to 2 weeks of hypoxia induces RV hypertrophy and numerous changes in gene expression in both the RV and LV. RNA‐seq (with on average, ~30 million 50 bp single reads) identified >16,000 genes expressed in both the RV and LV. Although we found many genes with DE between the RV_N_ and LV_N_ groups, the two ventricles showed less DE than occurred with hypoxia, in particular in the RV, which showed the largest number of increases or decreases in gene expression. We identified novel pathways enriched in the RV, as we will discuss below.

Enrichment of DE genes between the RV and LV in normoxia indicated unexpected differences in immune‐related genes. Pathways increased in the RV compared to the LV included “Innate Immune System,” “Complement Cascade,” “Immune System,” “Initial Triggering of Complement,” and “Regulation of Complement Cascade.” Thus, the right side of the heart, that is, the RV, may have a different immune “status” than the left side, in particular the LV. That idea is supported by our findings indicating a greater number of resident immune cells and expression of immune cell markers in the RV compared to the LV, as demonstrated by immunoblotting and flow cytometry.

The flow cytometric analysis revealed that the percentage of CD45^+^/CD11b/c^+^ cells in the RV was on average fourfold greater than that of the LV. This result suggests that the RV has a larger number of resident immune cells and may be more poised for certain immune responses than is the LV. STRING analysis on RNA‐seq data comparing the RV to the LV showed that the RV expressed a cluster of genes relating to antigen presentation, which further supports that the larger immune cell population in the RV is due to an increase in macrophages or dendritic cells, the two main professional antigen presenting cells. We speculate that such differences may contribute to the disproportionate fibrotic response (relative to what occurs in the LV) in the setting of RV hypertrophy, such as in pulmonary hypertension (Friedberg & Redington, 2014), since immune cells (e.g., macrophages) contribute to the generation of profibrotic myofibroblasts and subsequent fibrosis (Wynn & Ramalingam, 2012). Perhaps such cells contribute to the fibrosis found in the RV after hypoxia. Further work to define the immune cells and their function in the RV may reveal new insights regarding pathogenesis and therapies targeted to the RV. The pathway analysis of RNA‐seq data complemented these CD45^+^/CD11b/c^+^ results (Figure 3b and c) and suggested that the differentially expressed genes are associated with CD14^+^ monocytes, implying that the CD45^+^/CD11b/c^+^ cells are monocytes/macrophages rather than dendritic cells or other leukocytes.

Since hypoxia increased RV weight, it is perhaps not surprising that the largest number of genes with DE occurred between the RV_H_ and RV_N_ groups: 1,857 genes were significantly increased or decreased in expression. Enrichment analysis revealed that many of these genes relate to cell mitosis and proliferation, perhaps contributing to cardiac remodeling induced by hypoxia or changes in cell‐cycle and cell division‐related processes, associated with increased numbers of nonmyocytes (e.g., fibroblasts and immune cells). Additionally, we observed decreases in expression of gene groups related to catabolic processes including fatty acid catabolism, lipid oxidation, lipid modification and metabolic processes. Those findings are consistent with previous work showing altered metabolism in RV dysfunction (Ryan & Archer, 2014; Sharma et al., 2004) and imply that pathways involved in cardiac energy metabolism may contribute to changes in RV structure and function in disease.

Pathways and genes that are altered in both the RV and LV in response to hypoxia are of particular interest, as these shared changes may contribute to the cardiac response to hypoxia. Hypoxia mediates both cell autonomous and systemic changes, including endothelial signaling in the vasculature, which causes vasoconstriction in the systemic circulation but vasodilation in the pulmonary circulation. Shared genes altered in both ventricles during hypoxia may also result in the release of systemic factors such as circulating catecholamines (Ton & Hammes, 2014) that may cause similar responses via the shared coronary perfusion of both ventricles. Rat models of hypoxia exposure have been well‐characterized for physiological changes, and some studies have measured expression of key genes. However, it will be important to utilize other models of RV hypertrophy, such as pulmonary banding, to distinguish the gene expression changes related to changes in pressure and those related to hypoxia. Our results confirm previous work in rodent models of hypoxia, with respect to expression of fetal genes (*NPPA,* NPPB), fibrotic genes (*TGFB2, CTGF, LTBT2*), and genes marking cardiac hypertrophy (*NPPB, THBS4*) in the heart after exposure to hypoxia (Baertschi et al., 1986; Lew & Baertschi, 1989; Partovian et al., 1998; Toth et al., 1994). Our studies and analysis revealed that certain of these factors (notably, *NPPA* and *NPPB*) were increased in the RV compared to the LV even under normoxic conditions. This result suggests that the RV may be more primed for its response to hypoxia as compared to the LV.

The rat model of chronic hypoxia employed in this study is a reversible model of RV hypertrophy that occurs prior to decompensated RV failure (Nehra, Bhardwaj, Kar, & Saraswat, 2016). Thus, our findings reflect what is observed in the nonfailing RV. It will be of interest to identify the transcriptomic alterations that occur in RV failure, which occurs in response to pulmonary hypertension and also in the majority of left heart failure patients (Gulati et al., 2013; Mohammed et al., 2014; Puwanant et al., 2009). Recent work examining tissue from the RV of human heart failure patients found a set of genes that are unique to RV failure compared to LV failure: *WIPI1*, *HSPB6*, *SNAP47* and *MAP4* (Tzimas et al., 2019). These genes, which are altered in human and mouse RV failure, but not in less‐severe stages of mouse RV dysfunction (Tzimas et al., 2019), were not altered in our dataset (FDR > 0.05 RV normoxia vs. RV hypoxia). Those findings provide further evidence that 2 weeks of hypoxia yields RV dysfunction, but not RV failure. Similar work in human heart samples from patients with pulmonary arterial hypertension identified key genes that change uniquely in RV failure, including increased *FBN2* (which had a similar trend in our model), and CTGF (which was significantly increased in our model, FDR < 0.05). Additional work using microarrays to assess rat models of RV dysfunction and failure were in agreement with our findings of “markers” of RV dysfunction, including the increase in *ANGPT1*, *IFG1,* and no change in *ADH7* expression (Drake et al., 2011). Other studies have examined transcriptional signatures in similar models such as monocrotaline‐induced pulmonary hypertension in rats (Potus, Hindmarch, Dunham‐Snary, Stafford, & Archer, 2018), and surgically induced pulmonary insufficiency in mice (Reddy et al., 2013). Both models had similarities in gene group changes including cell cycle‐related genes, extracellular matrix organization, and decrease in metabolic pathways.

The data shown here were obtained from RV and LV tissue. Future studies will need to explore if the changes observed in normoxia and hypoxia result from altered expression in cardiac myocytes, cardiac fibroblasts or other nonmyocytes. We believe that the current results reveal numerous findings that merit further investigation to define hypoxia‐induced changes in the heart. This could be further examined by comparing the changes noted here with other models of RV hypertrophy and pulmonary hypertension. The transcriptomic data herein are a resource that may aid in identifying new aspects of pathobiology and perhaps, ultimately novel therapeutic approaches for the RV, perhaps including therapies directed at immune cells.

## CONFLICT OF INTEREST

The authors have no competing interests to declare.

## References

[phy214344-bib-0001] Afgan, E. , Baker, D. , van den Beek, M. , Blankenberg, D. , Bouvier, D. , Čech, M. , … Goecks, J. (2016). The Galaxy platform for accessible, reproducible and collaborative biomedical analyses: 2016 update. Nucleic Acids Research, 44, W3–W10. 27137889 10.1093/nar/gkw343 PMC4987906

[phy214344-bib-0002] Baertschi, A. J. , Hausmaninger, C. , Walsh, R. S. , Mentzer, R. M. , Wyatt, D. A. , & Pence, R. A. (1986). Hypoxia‐induced release of atrial natriuretic factor (ANF) from the isolated rat and rabbit heart. Biochemical and Biophysical Research Communications, 140, 427–433. 10.1016/0006-291X(86)91108-3 2946294

[phy214344-bib-0003] Binder, J. X. , Pletscher‐Frankild, S. , Tsafou, K. , Stolte, C. , O’Donoghue, S. I. , Schneider, R. , & Jensen, L. J. (2014). COMPARTMENTS: Unification and visualization of protein subcellular localization evidence. Database, 2014, bau012. 24573882 10.1093/database/bau012 PMC3935310

[phy214344-bib-0004] Bray, N. L. , Pimentel, H. , Melsted, P. , & Pachter, L. (2016). Near‐optimal probabilistic RNA‐seq quantification. Nature Biotechnology, 34, 525–527. 10.1038/nbt.3519 27043002

[phy214344-bib-0005] Chun, Y.‐S. , Hyun, J.‐Y. , Kwak, Y.‐G. , Kim, I.‐S. , Kim, C.‐H. , Choi, E. , … Park, J.‐W. (2003). Hypoxic activation of the atrial natriuretic peptide gene promoter through direct and indirect actions of hypoxia‐inducible factor‐1. Biochemical Journal, 370, 149–157. 10.1042/bj20021087 12413399 PMC1223144

[phy214344-bib-0006] Drake, J. I. , Bogaard, H. J. , Mizuno, S. , Clifton, B. , Xie, B. , Gao, Y. , … Natarajan, R. (2011). Molecular signature of a right heart failure program in chronic severe pulmonary hypertension. American Journal of Respiratory Cell and Molecular Biology, 45, 1239–1247. 10.1165/rcmb.2010-0412OC 21719795 PMC3361357

[phy214344-bib-0007] Fabregat, A. , Jupe, S. , Matthews, L. , Sidiropoulos, K. , Gillespie, M. , Garapati, P. , … D’Eustachio, P. (2018). The reactome pathway knowledgebase. Nucleic Acids Research, 46, D649–D655. 10.1093/nar/gkx1132 29145629 PMC5753187

[phy214344-bib-0008] Friedberg, M. K. , & Redington, A. N. (2014). Right versus left ventricular failure: Differences, similarities, and interactions. Circulation, 129, 1033–1044. 24589696 10.1161/CIRCULATIONAHA.113.001375

[phy214344-bib-0009] Ghio, S. , Gavazzi, A. , Campana, C. , Inserra, C. , Klersy, C. , Sebastiani, R. , … Tavazzi, L. (2001). Independent and additive prognostic value of right ventricular systolic function and pulmonary artery pressure in patients with chronic heart failure. Journal of the American College of Cardiology, 37, 183–188. 10.1016/S0735-1097(00)01102-5 11153735

[phy214344-bib-0010] Gulati, A. , Ismail, T. F. , Jabbour, A. , Alpendurada, F. , Guha, K. , Ismail, N. A. , … Prasad, S. K. (2013). The Prevalence and prognostic significance of right ventricular systolic dysfunction in nonischemic dilated cardiomyopathy. Circulation, 128, 1623–1633. 10.1161/CIRCULATIONAHA.113.002518 23965488

[phy214344-bib-0011] Kuleshov, M. V. , Jones, M. R. , Rouillard, A. D. , Fernandez, N. F. , Duan, Q. , Wang, Z. , … Ma’ayan, A. (2016). Enrichr: A comprehensive gene set enrichment analysis web server 2016 update. Nucleic Acids Research, 44, W90–W97. 27141961 10.1093/nar/gkw377 PMC4987924

[phy214344-bib-0012] Lettieri, C. J. , Nathan, S. D. , Barnett, S. D. , Ahmad, S. , & Shorr, A. F. (2006). Prevalence and outcomes of pulmonary arterial hypertension in advanced idiopathic pulmonary fibrosis. Chest, 129, 746–752. 10.1378/chest.129.3.746 16537877

[phy214344-bib-0013] Lew, R. A. , & Baertschi, A. J. (1989). Mechanisms of hypoxia‐induced atrial natriuretic factor release from rat hearts. American Journal of Physiology‐Heart and Circulatory Physiology, 257, H147–H156. 10.1152/ajpheart.1989.257.1.H147 2526588

[phy214344-bib-0014] Meyer, P. , Filippatos, G. S. , Ahmed, M. I. , Iskandrian, A. E. , Bittner, V. , Perry, G. J. , … Ahmed, A. (2010). Effects of right ventricular ejection fraction on outcomes in chronic systolic heart failure. Circulation, 121, 252–258. 10.1161/CIRCULATIONAHA.109.887570 20048206 PMC2877272

[phy214344-bib-0015] Mi, H. , Muruganujan, A. , Ebert, D. , Huang, X. , & Thomas, P. D. (2019). PANTHER version 14: More genomes, a new PANTHER GO‐slim and improvements in enrichment analysis tools. Nucleic Acids Research, 47, D419–D426. 30407594 10.1093/nar/gky1038 PMC6323939

[phy214344-bib-0016] Mohammed, S. F. , Hussain, I. , AbouEzzeddine, O. F. , Takahama, H. , Kwon, S. H. , Forfia, P. , … Redfield, M. M. (2014). Right ventricular function in heart failure with preserved ejection fraction. Circulation, 130, 2310–2320. 10.1161/CIRCULATIONAHA.113.008461 25391518 PMC4276536

[phy214344-bib-0017] Nehra, S. , Bhardwaj, V. , Kar, S. , & Saraswat, D. (2016). Chronic hypobaric hypoxia induces right ventricular hypertrophy and apoptosis in rats: Therapeutic potential of nanocurcumin in improving adaptation. High Altitude Medicine & Biology, 17, 342–352. 27626325 10.1089/ham.2016.0032

[phy214344-bib-0018] Partovian, C. , Adnot, S. , Eddahibi, S. , Teiger, E. , Levame, M. , Dreyfus, P. , … Frelin, C. (1998). Heart and lung VEGF mRNA expression in rats with monocrotaline‐ or hypoxia‐induced pulmonary hypertension. American Journal of Physiology‐Heart and Circulatory Physiology, 275, H1948–H1956. 10.1152/ajpheart.1998.275.6.H1948 9843792

[phy214344-bib-0019] Potus, F. , Hindmarch, C. , Dunham‐Snary, K. , Stafford, J. , & Archer, S. (2018). Transcriptomic signature of right ventricular failure in experimental pulmonary arterial hypertension: Deep sequencing demonstrates mitochondrial, fibrotic, inflammatory and angiogenic abnormalities. The International Journal of Molecular Sciences, 19, 2730. 10.3390/ijms19092730 PMC6164263 30213070

[phy214344-bib-0020] Puwanant, S. , Priester, T. C. , Mookadam, F. , Bruce, C. J. , Redfield, M. M. , & Chandrasekaran, K. (2009). Right ventricular function in patients with preserved and reduced ejection fraction heart failure. European Journal of Echocardiography, 10, 733–737. 10.1093/ejechocard/jep052 19443468

[phy214344-bib-0021] Reddy, S. , Zhao, M. , Hu, D.‐Q. , Fajardo, G. , Katznelson, E. , Punn, R. , … Bernstein, D. (2013). Physiologic and molecular characterization of a murine model of right ventricular volume overload. American Journal of Physiology‐Heart and Circulatory Physiology, 304, H1314–H1327. 10.1152/ajpheart.00776.2012 23504182 PMC3652064

[phy214344-bib-0022] Robinson, M. D. , McCarthy, D. J. , & Smyth, G. K. (2010). edgeR: A Bioconductor package for differential expression analysis of digital gene expression data. Bioinformatics, 26, 139–140. 19910308 10.1093/bioinformatics/btp616 PMC2796818

[phy214344-bib-0023] Ryan, J. J. , & Archer, S. L. (2014). The right ventricle in pulmonary arterial hypertension: Disorders of metabolism, angiogenesis and adrenergic signaling in right ventricular failure. Circulation Research, 115, 176–188. 24951766 10.1161/CIRCRESAHA.113.301129 PMC4112290

[phy214344-bib-0024] Sharma, S. , Taegtmeyer, H. , Adrogue, J. , Razeghi, P. , Sen, S. , Ngumbela, K. , & Essop, M. F. (2004). Dynamic changes of gene expression in hypoxia‐induced right ventricular hypertrophy. American Journal of Physiology‐Heart and Circulatory Physiology, 286, H1185–H1192. 10.1152/ajpheart.00916.2003 14630626

[phy214344-bib-0025] Soneson, C. , Love, M. I. , & Robinson, M. D. (2015). Differential analyses for RNA‐seq: Transcript‐level estimates improve gene‐level inferences. F1000Research, 4, 1521. 26925227 10.12688/f1000research.7563.1 PMC4712774

[phy214344-bib-0026] Subramanian, A. , Tamayo, P. , Mootha, V. K. , Mukherjee, S. , Ebert, B. L. , Gillette, M. A. , … Mesirov, J. P. (2005). Gene set enrichment analysis: A knowledge‐based approach for interpreting genome‐wide expression profiles. Proceedings of the National Academy of Sciences of the United States of America, 102, 15545–15550. 16199517 10.1073/pnas.0506580102 PMC1239896

[phy214344-bib-0027] Szklarczyk, D. , Franceschini, A. , Wyder, S. , Forslund, K. , Heller, D. , Huerta‐Cepas, J. , … von Mering, C. (2015). STRING v10: Protein–protein interaction networks, integrated over the tree of life. Nucleic Acids Research, 43, D447–D452. 25352553 10.1093/nar/gku1003 PMC4383874

[phy214344-bib-0028] Terzano, C. , Conti, V. , Di Stefano, F. , Petroianni, A. , Ceccarelli, D. , Graziani, E. , … Allegra, L. (2010). Comorbidity, hospitalization, and mortality in COPD: Results from a longitudinal study. Lung, 188, 321–329. 20066539 10.1007/s00408-009-9222-y

[phy214344-bib-0029] Ton, Q. V. , & Hammes, S. R. (2014). Recent insights on circulating catecholamines in hypertension. Current Hypertension Reports, 16, 498 10.1007/s11906-014-0498-9 25304108

[phy214344-bib-0030] Toth, M. , Vuorinen, K. H. , Vuolteenaho, O. , Hassinen, I. E. , Uusimaa, P. A. , Leppaluoto, J. , & Ruskoaho, H. (1994). Hypoxia stimulates release of ANP and BNP from perfused rat ventricular myocardium. American Journal of Physiology‐Heart and Circulatory Physiology, 266, H1572–H1580. 10.1152/ajpheart.1994.266.4.H1572 8184936

[phy214344-bib-0031] Tzimas, C. , Rau, C. D. , Buergisser, P. E. , Jean‐Louis, G. , Lee, K. , Chukwuneke, J. , … Tsai, E. J. (2019). WIPI1 is a conserved mediator of right ventricular failure. JCI Insight, 4(11), e122929 10.1172/jci.insight.122929 PMC6629151 31021818

[phy214344-bib-0032] Wu, C. , Orozco, C. , Boyer, J. , Leglise, M. , Goodale, J. , Batalov, S. , … Su, A. I. (2009). BioGPS: An extensible and customizable portal for querying and organizing gene annotation resources. Genome Biology, 10, R130. 19919682 10.1186/gb-2009-10-11-r130 PMC3091323

[phy214344-bib-0033] Wynn, T. A. , & Ramalingam, T. R. (2012). Mechanisms of fibrosis: Therapeutic translation for fibrotic disease. Nature Medicine, 18, 1028–1040. 10.1038/nm.2807 PMC3405917 22772564

