# Supplementary figures and images for: Transcriptomic profiles reveal differences between the right and left ventricle in normoxia and hypoxia
Source: Physiol Rep. 2020 Jan 20;8(2):e14344. doi: 10.14814/phy2.14344 (PMC6971333; doi:10.14814/phy2.14344)

### Figure S1

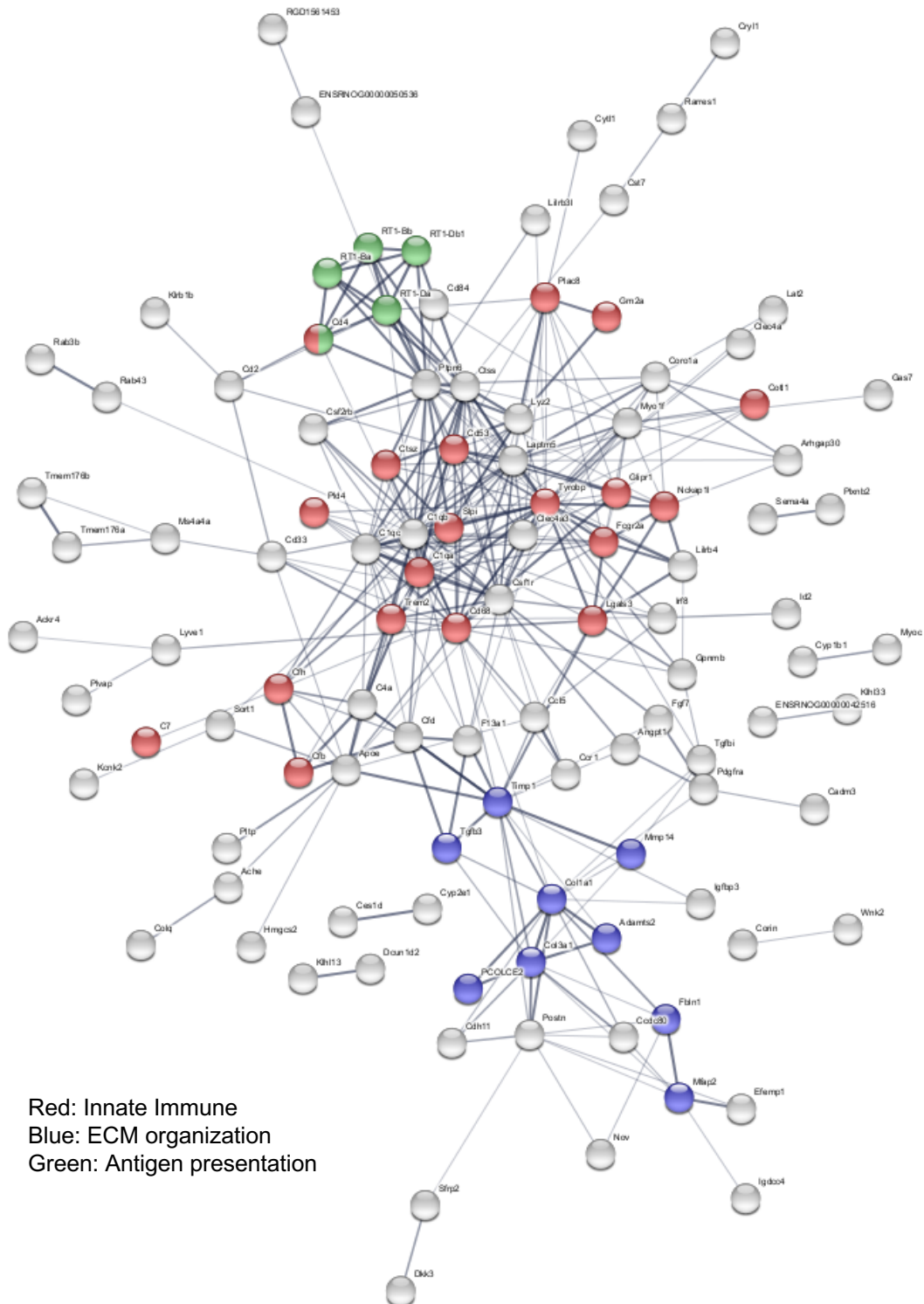

Supplement: Supplementary file 1 [file PHY2-8-e14344-s001.pdf]

### Figure S2

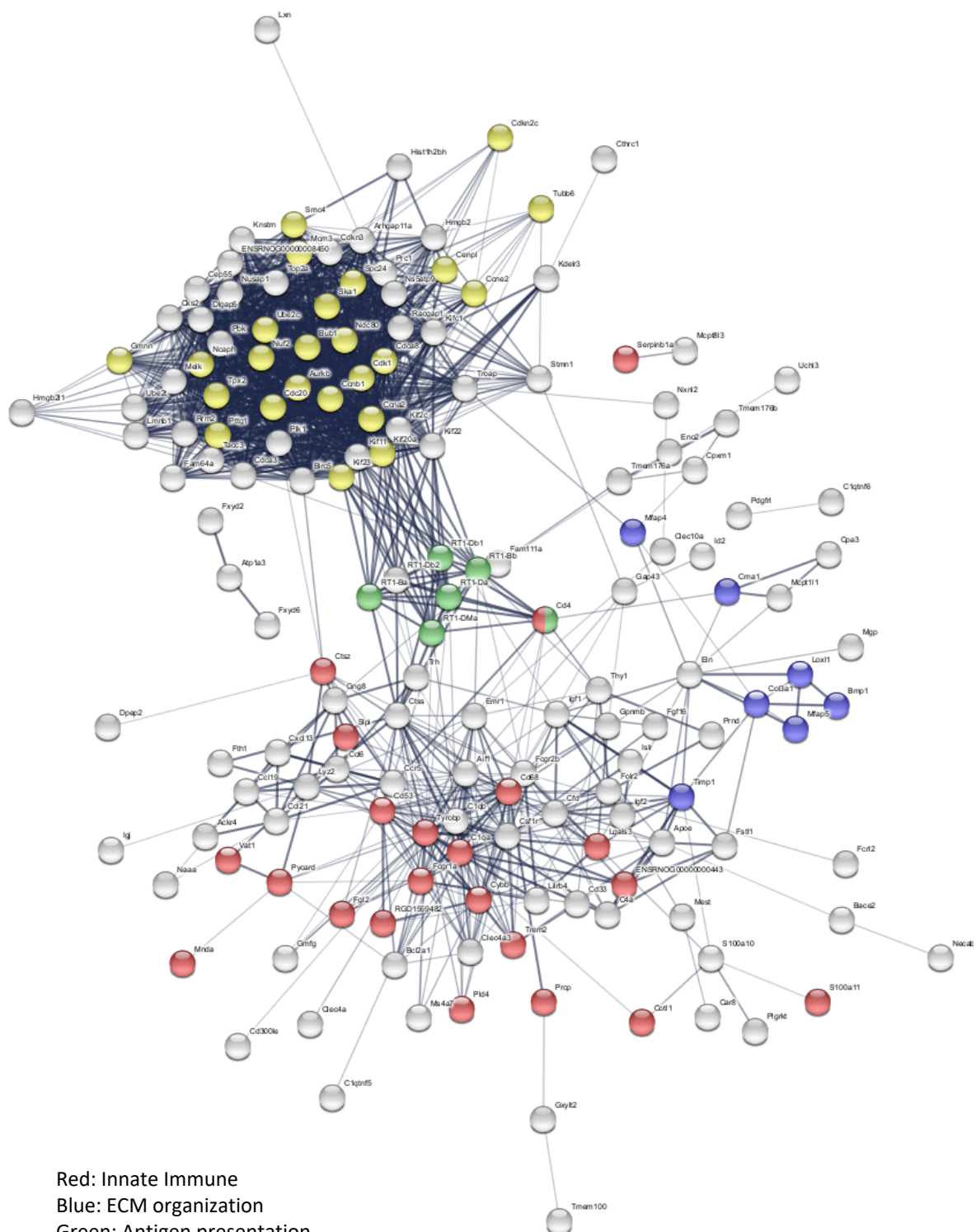

Supplement: Supplementary file 2 [file PHY2-8-e14344-s002.pdf]
